# Directed evolution of single-chain Fv for cytoplasmic expression using the β-galactosidase complementation assay results in proteins highly susceptible to protease degradation and aggregation

**DOI:** 10.1186/1475-2859-3-16

**Published:** 2004-12-17

**Authors:** Pascal Philibert, Pierre Martineau

**Affiliations:** 1 CNRS UMR 5160, Faculté de Pharmacie, 15, av. Charles Flahault, BP14491, 34093. Montpellier Cedex 5, France

## Abstract

**Background:**

Antibody fragments are molecules widely used for diagnosis and therapy. A large amount of protein is frequently required for such applications. New approaches using folding reporter enzymes have recently been proposed to increase soluble expression of foreign proteins in *Escherichia coli*. To date, these methods have only been used to screen for proteins with better folding properties but have never been used to select from a large library of mutants. In this paper we apply one of these methods to select mutations that increase the soluble expression of two antibody fragments in the cytoplasm of *E. coli*.

**Results:**

We used the β-galactosidase α-complementation system to monitor and evolve two antibody fragments for high expression levels in *E. coli *cytoplasm. After four rounds of mutagenesis and selection from large library repertoires (>10^7 ^clones), clones exhibiting high levels of β-galactosidase activity were isolated. These clones expressed a higher amount of soluble fusion protein than the wild type in the cytoplasm, particularly in a strain deficient in the cytoplasmic Lon protease. The increase in the soluble expression level of the unfused scFv was, however, much less pronounced, and the unfused proteins proved to be more aggregation prone than the wild type. In addition, the soluble expression levels were not correlated with the β-galactosidase activity present in the cells.

**Conclusion:**

This is the first report of a selection for soluble protein expression using a fusion reporter method. Contrary to anticipated results, high enzymatic activity did not correlate with the soluble protein expression level. This was presumably due to free α-peptide released from the protein fusion by the host proteases. This means that the α-complementation assay does not sense the fusion expression level, as hypothesized, but rather the amount of free released α-peptide. Thus, the system does not select, in our case, for higher soluble protein expression level but rather for higher protease susceptibility of the fusion protein.

## Background

Because of their high affinity and specificity against their antigen, antibody molecules and their fragments have many applications in research, diagnosis and therapy [1]. *E. coli *is a widely used organism for the production of proteins, including antibody fragments such as Fab or single chain variable domains (scFv). Active scFv can be obtained by targeting the protein to *E. coli *periplasm, where the two disulfide bonds needed for protein folding and stability [2-4] can form. The amount of scFv produced is however usually low, in the range of 0.1–1 mg l^-1 ^of culture at an optical density (OD_600_) of 1, even if expression levels of 10 mg l^-1 ^have sometimes been reported [5].

An alternative strategy to produce antibody fragments in *E. coli *has been to maintain the scFv in the cytoplasm by removing its signal sequence. Under those conditions, scFv might be expressed at very high levels, albeit in an insoluble and inactive conformation. Even if highly efficient *in vitro *refolding procedures have been developed for scFv and Fab [6,7], it would be more suitable to directly recover soluble active protein from the cytoplasm of the cell. This has been partially accomplished by modifying the cytoplasmic oxido-redox environment by mutating components of the thioredoxin and glutaredoxin pathways [8], resulting in the accumulation of soluble intra-cytoplasmic oxidized antibody fragments [9-11]. The soluble expression levels reported, however, are not higher than those obtained in the periplasm of the cell. Another interest in expressing soluble active scFv in the reducing environment of the cytoplasm is that the expression of scFv molecules inside the cell can be used to block viral replication and to inhibit oncogene products [12-15]. The use of so-called intrabodies opens many interesting possibilities in gene therapy [16] and in the *in vivo *study of protein function [17].

Several methods have been proposed in recent years to increase the soluble expression levels of foreign proteins expressed in *E. coli *cytoplasm [18]. Most of these methods rely on fusion between the protein of interest and a reporter enzyme. If the protein folds into a soluble conformation, the fused enzyme will be active; but if the protein ends up as an inclusion body, the fused enzyme will be inactive, resulting in a null phenotype. Three reporter enzymes have been used to date, the green fluorescent protein (GFP [19]), the chloramphenicol acetyl transferase (CAT [20]) and the β-galactosidase (βgal [21]). In the case of βgal, the whole enzyme was not fused to the protein of interest but only a small N-terminal fragment of about 50 aminoacids called the α-fragment [22]. The inactive enzyme remainder was expressed in *trans *by the bacteria (β-galactosidase ΔM15 protein), resulting in *in vivo *complementation and a lactose+ (lac+) phenotype [23]. The role of the α-fragment is to promote the tetramerization of the inactive dimeric ΔM15 mutant [24], resulting in βgal activation.

Using the GFP fusion method, Waldo and collaborators [18,25,26] isolated several soluble variants of aggregating proteins. However, since the method is based on a phenotypic screen, it is limited to the exploration of libraries of about 10^5 ^clones and does not allow the isolation of mutants from very large libraries. This is not the case for the two other methods which should allow the selection of very rare events by selecting for chloramphenicol resistance or growth on lactose as the carbon source. To date, there is, however, no report of protein evolution and selection using these latter two methods even if the CAT system has been used to pre-select libraries of hybrid proteins to avoid stop codons and enrich in properly folded molecules [27].

We previously showed that it is possible to evolve an scFv molecule for very high expression levels in *E. coli *cytoplasm [4,28,29]. This evolved scFv is active in the cell and its expression level is as high as 100 mg l^-1 ^at an OD_600 _of 1. This was, however, a very rare event, and the selection had to be conducted using large libraries of 10^7^–10^8 ^mutants. The selection procedure used was restricted to the properties of a particular antibody molecule able to activate βgal mutants *in vitro *and *in vivo*. To extend this result to other scFv molecules, we constructed fusions between two scFv and the α-fragment of βgal to monitor the soluble expression level *in vivo*. The lactose phenotype of the strain was then used to select mutants with improved lactose utilization and thus presumably expressing scFv at a higher level in the bacterial cytoplasm.

## Results

### System design

**Figure 1:**
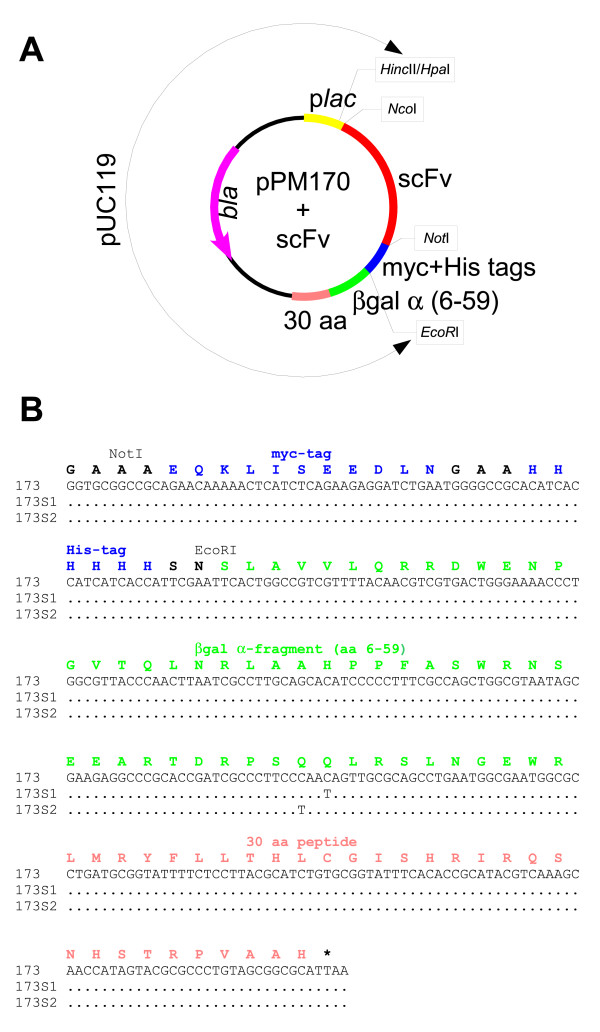
**Schematic view of plasmid pPM170 **Plasmid pPM170 allows the cloning of scFv genes between the *Nco*I and *Not*I sites under the control of the lactose promoter. The translated protein is fused at its C-terminal extremity to the α-fragment of βgal. A) Plasmid map with the main restriction sites; B) DNA and aminoacid sequences fused to the 3' end and the C-terminus of the cloned scFv. The scFv sequence is followed by two tags (in blue), the α-fragment of βgal (in green, aminoacids 6 to 59 of βgal) and a 30 aminoacid long peptide (in orange) originating from pBR322 plasmid and M13 gene IV sequence (see text). The sequences of the two sequenced spontaneous lac+ mutants, 173S1 and 173S2 (see text), are also shown.

In order to easily fuse scFv to the α-fragment of βgal, we constructed the plasmid pPM170, derived from pUC119 and which contains a *lac *promoter followed by a *Nco*I and a *Not*I site in which an scFv can be easily cloned in frame with the α-fragment present in the pUC119 plasmid. The α-fragment is separated from the protein by a linker consisting of two tags (Fig. 1).

**Figure 2:**
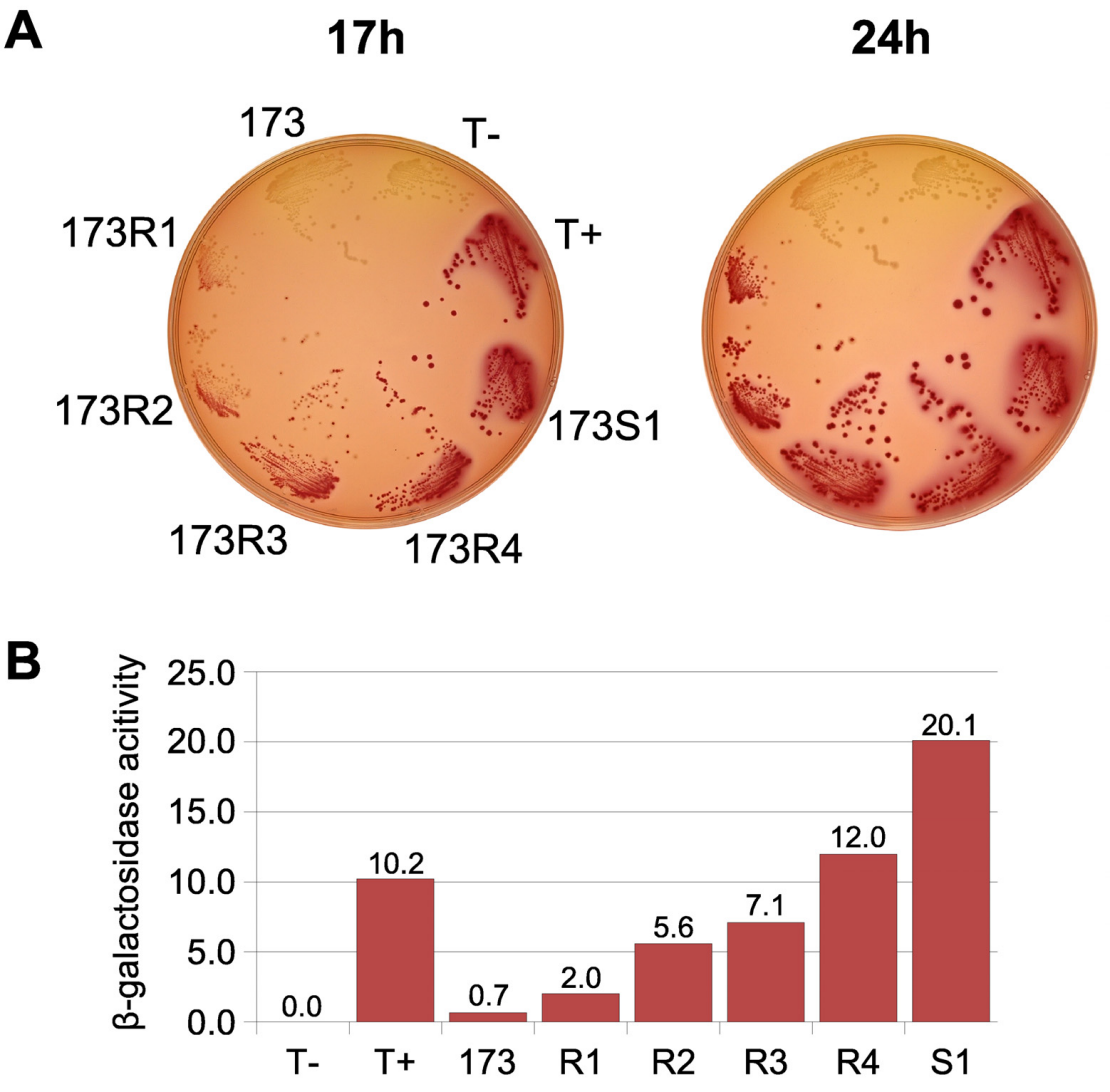
**Lactose phenotype correlates with the soluble expression levels **Phenotype of strain TG1 transformed with the pPM170 plasmid containing a series of scFv with different soluble expression levels in *E. coli *cytoplasm. The scFv13, 13R1, 13R2, 13R3 and 13R4 are described in [28]. T- and T+ are TG1 transformed with the pTrc99A and pUC119 plasmids, respectively. 173S1 is a spontaneous lac+ mutant isolated from TG1(pPM173) (see text and Fig. 1). A) Lactose phenotype on MacConkey lactose plates. The ability of the cell to use lactose is proportional to the depth of the red coloration. B) βgal activity present in the cytoplasm of the cells measured using Miller's whole cell assay [30].

To verify that the system was indeed able to discriminate soluble scFv expression levels in the cytoplasm, we used a set of mutant scFv derived from the human scFv13 and presenting a gradual increase in soluble expression [28]. The expression levels of these scFv have been previously studied in *E. coli *cytoplasm using the tryptophan promoter: The best mutant, 13R4, is expressed at about a 50 times higher level than the wild type scFv13 and the expression levels increased gradually from scFv13 to scFv13R4 (in the rank order 13 < 13R1 < 13R2 < 13R3 < 13R4). These scFv were cloned in pPM170 (plasmids pPM173 to pPM173R4). Fig. 2A shows the phenotype on MacConkey lactose plates of strain TG1 containing the plasmids. As expected, the phenotype gradually increased from lac- (white colonies) for pPM173 expressing the wild type scFv13 to a strong lac+ phenotype when the plasmid contained the best expressed mutant 173R4. The phenotype of this latter strain is comparable to the phenotype of the strain containing an unmodified pUC119 plasmid giving normal α-complementation (T+).

The level of α-complementation was further characterized by measuring the βgal activity present in the cell using the whole cell assay developed by Miller [30]. The result shown in Fig. 2B demonstrated that the βgal activity in TG1(pPM173) was close to background level and that the activity increased gradually to 100% of the activity of the positive α-complementation control. This clearly demonstrated the correlation between the soluble cytoplasmic expression levels of the scFv and the lactose phenotype of the strain, opening the way to direct selection of mutant proteins using this assay.

### Spontaneous lactose plus mutants

When TG1(pPM173) was incubated 2 to 3 days at 37°C, red papillae appeared on the MacConkey plates. These clones were isolated and showed a stable lac+ phenotype, comparable to a wild type lac+ strain. One of the clones, 173S1, had even a higher βgal activity in its cytoplasm than the pUC119 rescued ΔM15 strain (Fig. 2B). The increase in βgal activity was shown to be linked to the plasmid by transforming TG1 with the plasmid extracted from TG1(pPM173S1). To determine if the mutation was located in the scFv, we excised the gene from the plasmid using the *Nco*I and *Not*I sites and cloned it back in pPM170. The resulting clone was lac- (data not shown), demonstrating that the mutation responsible for the lac+ phenotype was associated with the plasmid but not with the scFv.

To understand the origin of this phenotypic change, we sequenced the α-fragment of two of the clones, 173S1 and 173S2. Both contained a mutation in the α-fragment of the βgal, resulting in a stop codon (Fig. 1) at positions 49 and 50. It should be noted that the stop codon of 173S1 is an amber codon which is partially suppressed in TG1 and replaced by a Gln, resulting in this case in two different fusions, one stopping at position 49 of the βgal and the second with a complete α-fragment, showing that the mutant is dominant over the wild type.

Analysis of the sequence of the α-fragment present in pUC119 (and pPM170), showed that a 30 aminoacid long peptide of unknown origin was fused to the C-terminal extremity of the α-fragment (Fig. 1). This peptide (or part of it) was present in all the pUC derived plasmids. Sequence analysis showed that this peptide originated from a fusion between a DNA sequence present in pBR322 plasmid between the *Nde*I and *Ear*I sites (nucleotides 2297 to 2351 of pBR322; LMR...HRI translated sequence) and the 3' end of M13 gene IV (nucleotides 5470 to 5511 of M13; RQS...AAH translated sequence). The pBR322 sequence, located between the *rop *gene and the origin of replication, is not normally translated, and the M13 gene IV sequence is read out of frame.

**Figure 3:**
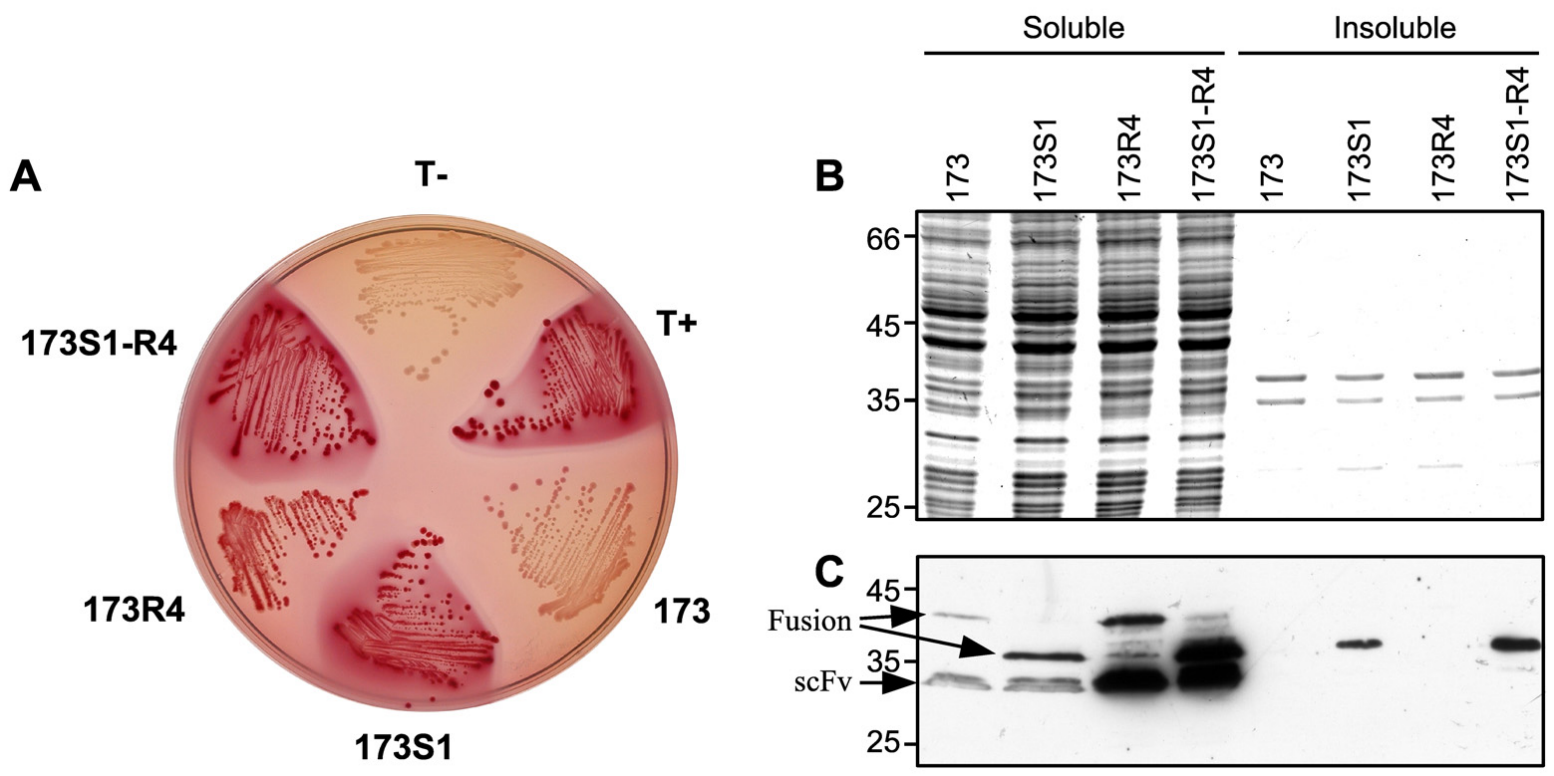
**Effect of the 30 aminoacid long C-terminal peptide on scFv expression level **scFv13 and scFv13R4 were cloned either in pPM170 (173 and 173R4) or in pPM173S1 (173S1 and 173S1-R4), and expressed in strain TG1. A) Phenotype of the strains on MacConkey lactose plates after 24 h at 37°C. B) Soluble and insoluble extracts of the four strains were analyzed by Coomassie blue staining (top) or by western blot (bottom) using the 9E10 anti-*c*-myc monoclonal antibody followed by an anti-mouse HRP-conjugated antibody and detected with a commercial kit (Pierce Supersignal West Pico kit #14079). The arrows show the position of the fusions (38 kDa for 173 and 173R4; 34 kDa for 173S1 and 173S1-R4) and the unfused scFv (28 kDa).

Two explanations could account for the stronger lactose phenotype of the 173S1 clone when the extra peptide was removed from the fusion. Either this peptide impaired the α-complementation, or the presence of this peptide changed the expression level of the scFv. To test these hypotheses, we expressed the scFv13 and scFv13R4 clones as fusion proteins either to the α-peptide followed by the 30 aminoacid long peptide (173 and 173R4) or to the shorter α-peptide of clone 173S1 (173S1 and 173S1-R4). As shown in Figure 3B, the expression level of the fusions was much higher with the shorter α-peptide. This demonstrated that the additional C-terminal peptide lowered the protein expression level, presumably by directing the scFv to the cell degradation machinery since both the insoluble and the soluble expression levels where decreased. In all cases, the fusion proteins were degraded, and a band migrating to about 30 kDa was visible. This band may correspond to an unfused scFv liberated by host proteases.

However, despite a strong increase in the expression level of 173S1-R4 versus 173S1, the lactose phenotypes of both clones expressed in strain TG1 were comparable (Fig. 3A). This showed that the link between the lactose phenotype and the intracellular expression levels of scFv13 shown in Figure 2 was due to the presence of the 30 aminoacid long peptide. Indeed, in the case of 173S1, the α-complementation detection system is saturated and the strong lactose phenotype of strain TG1(173S1) would not allow the selection of evolved mutants with a stronger phenotype. This prompted us to retain this foreign 30 aminoacid long peptide for selection and to adapt the procedure to avoid the selection of spontaneous stop codons.

### Molecular evolution

**Figure 4:**
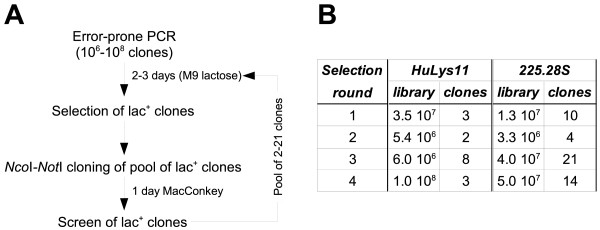
**Outline of the selection procedure **A) Outline of the steps followed during the selection procedure; B) Size of the libraries generated and number of clones isolated after each round of selection and used as a pool for the next round.

The outline of the selection procedure is shown in Figure 4A. We first introduced random mutations at a low rate (0.2%) in the scFv gene using error-prone PCR. The mutagenized gene was cloned in the pPM170 vector and transformed in TG1 bacteria to give a library of at least 10^7 ^clones. Transformed bacteria were plated on selective medium containing lactose as the unique carbon source, allowing the selection of lac+ clones. After 1 to 3 days at 37°C, about 10 to 100 clones able to grow under these conditions were cultured in 96-well microtiter plates, then pooled before plasmid extraction. To avoid the isolation of spontaneous lac+ mutations in the α-fragment (see above), the *Nco*I-*Not*I fragment containing the scFv gene was excised from the pooled plasmids and cloned back in pPM170. After transformation in TG1, a small number of clones (typically 100 to 1000) was screened on MacConkey lactose agar plates and the deepest red colonies further analyzed. This last screen was done after a 24-h incubation at 37°C in order to avoid the selection of spontaneous red colonies that appeared after about 2 to 3 days.

Two scFv were chosen as models. We first used a humanized D1.3 anti-lysozyme antibody, HuLys11 [31]. This antibody has been extensively studied and its structure solved by X-ray crystallography. The second scFv was derived from the mouse monoclonal antibody 225.28S and recognizes the high molecular weight melanoma-associated antigen [32]. As such, this antibody might have potential applications in the treatment of that type of cancer.

**Figure 5:**
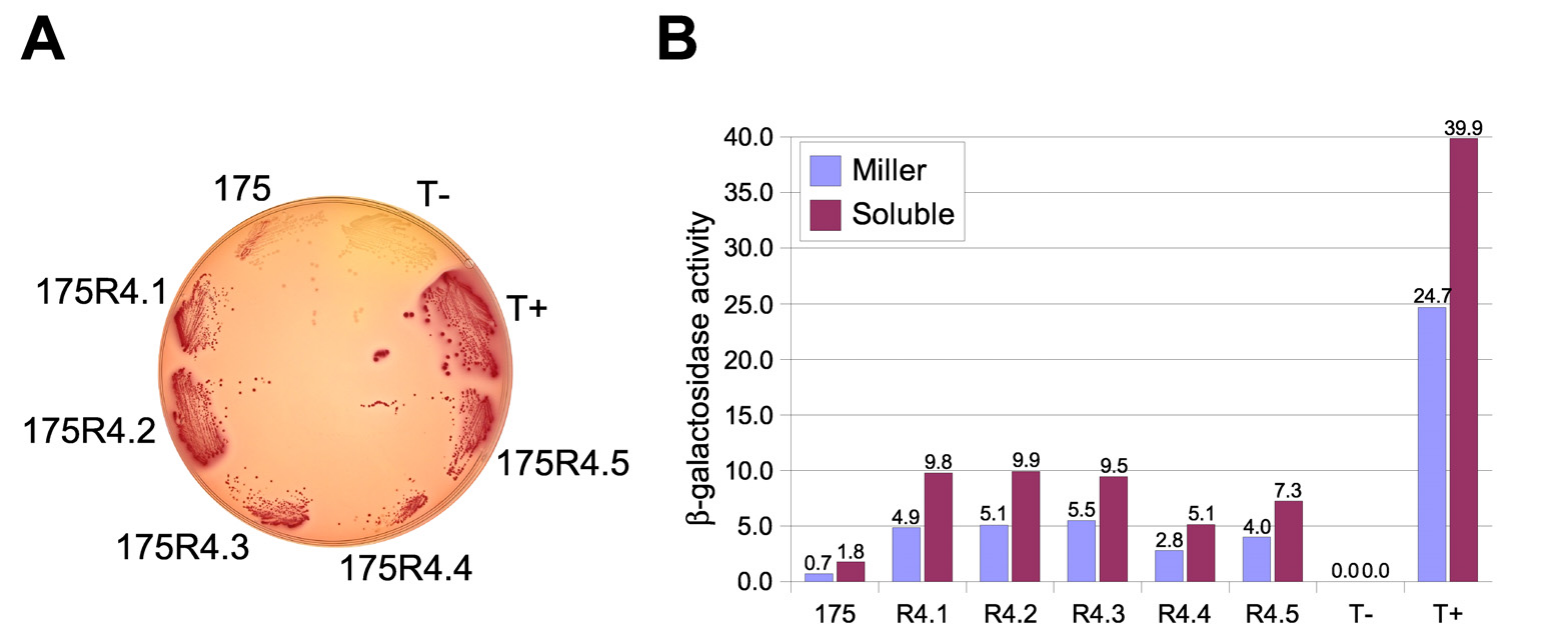
**Phenotype of the isolated mutants **After four rounds of mutagenesis and selection, the five best clones of scFv 225.28S were analyzed in strain TG1. pPM175 contains the wild type scFv and pPM175R4.1 to pPM175R4.5 the five isolated mutants. T+ and T- are the same as in Fig. 2. A) Phenotype of the strains on MacConkey lactose plates; B) βgal activity measured as in Figure 1. In addition to the Miller's whole cell assay (blue), we analyzed in B the βgal activity present in soluble extracts (red).

Four rounds of mutagenesis and selection were done for both scFv. The size of the libraries and the number of clones isolated after each round are shown in Figure 4B. The phenotype of the clones increased during the selection from an almost lac- to a clearly lac+ phenotype. This is shown in Figure 5A for 225.28S scFv, where the best five isolated clones (R4.1 to R4.5) exhibited a deeper red color than the original clone (175), demonstrating a greater ability to use lactose as a carbon source. In addition, the phenotype was close to the phenotype obtained with the pUC119 plasmid, that is, normal α-complementation. The results obtained with HuLys11 were comparable, but the colonies showed a weaker lactose phenotype (data not shown).

**Figure 6:**
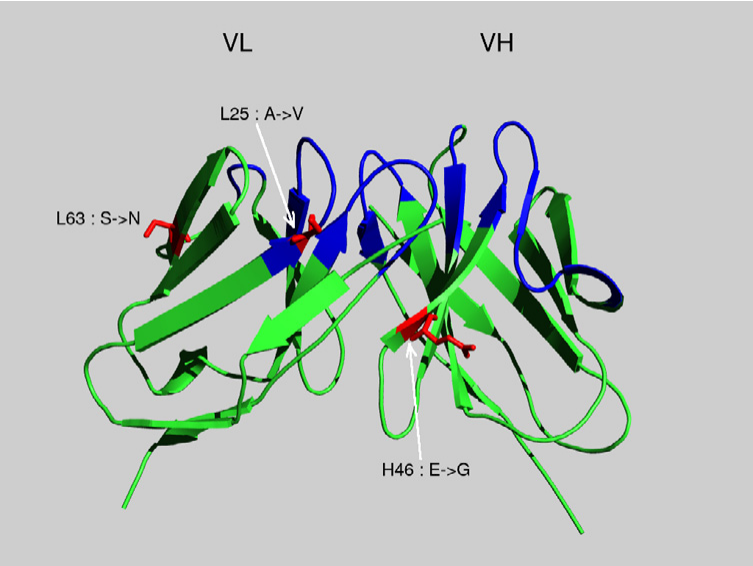
**Location of isolated mutations in HuLys11 scFv structure **Schematic representation of the HuLys11 scFv (pdb 1BVK). The CDR loops are represented in blue. The side chain of three of the four isolated mutations (see additional file HuLys11aa.txt) are represented in red. The fourth mutation, located in the linker sequence, is not present in the structure.

All the isolated clones were different at the DNA and protein level. The mutants of scFv225.28S contained 8–14 nucleotide substitutions and 3–9 mutations in the protein (see additional files 1 and 2, 22528Sdna.txt and 22528Saa.txt). In the case of HuLys11, the three isolated mutants contained 4–5 nucleotide substitutions and 2–3 aminoacid mutations (see additional files 3 and 4, HuLys11dna.txt and HuLys11aa.txt). The mutations did not cluster together either in the DNA sequence nor in the protein or in the structure of the scFv. In the case of HuLys11, for which a high resolution structure is available, three of the four isolated mutations are located in β-strands and are solvent exposed (see additional file 4, HuLys11aa.txt and Fig. 6).

To confirm the phenotype shown on the plates, βgal activity was measured using the assay described by Miller [30]. The assay relies on cell lysis by chloroform/SDS and as such measures the amount of βgal activity present in the whole cell. As shown in Figure 5B, the βgal activity present in the mutants was about five to seven times higher than in the strain expressing the wild type 225.28S scFv fused to the α-fragment and five times lower than in the control cells expressing the unfused α-fragment from the pUC119 plasmid. In oder to confirm that this increase in βgal activity was localized in the soluble protein fraction, we measured the activity in soluble extracts of the cells prepared by lysozyme treatment and centrifugation. The results were comparable to those obtained with the whole cell assay (Fig. 5B), demonstrating that indeed the increase in βgal activity was due to an increase in α-complementation in the soluble protein fraction.

### Fused scFv-α characterization

**Figure 7:**
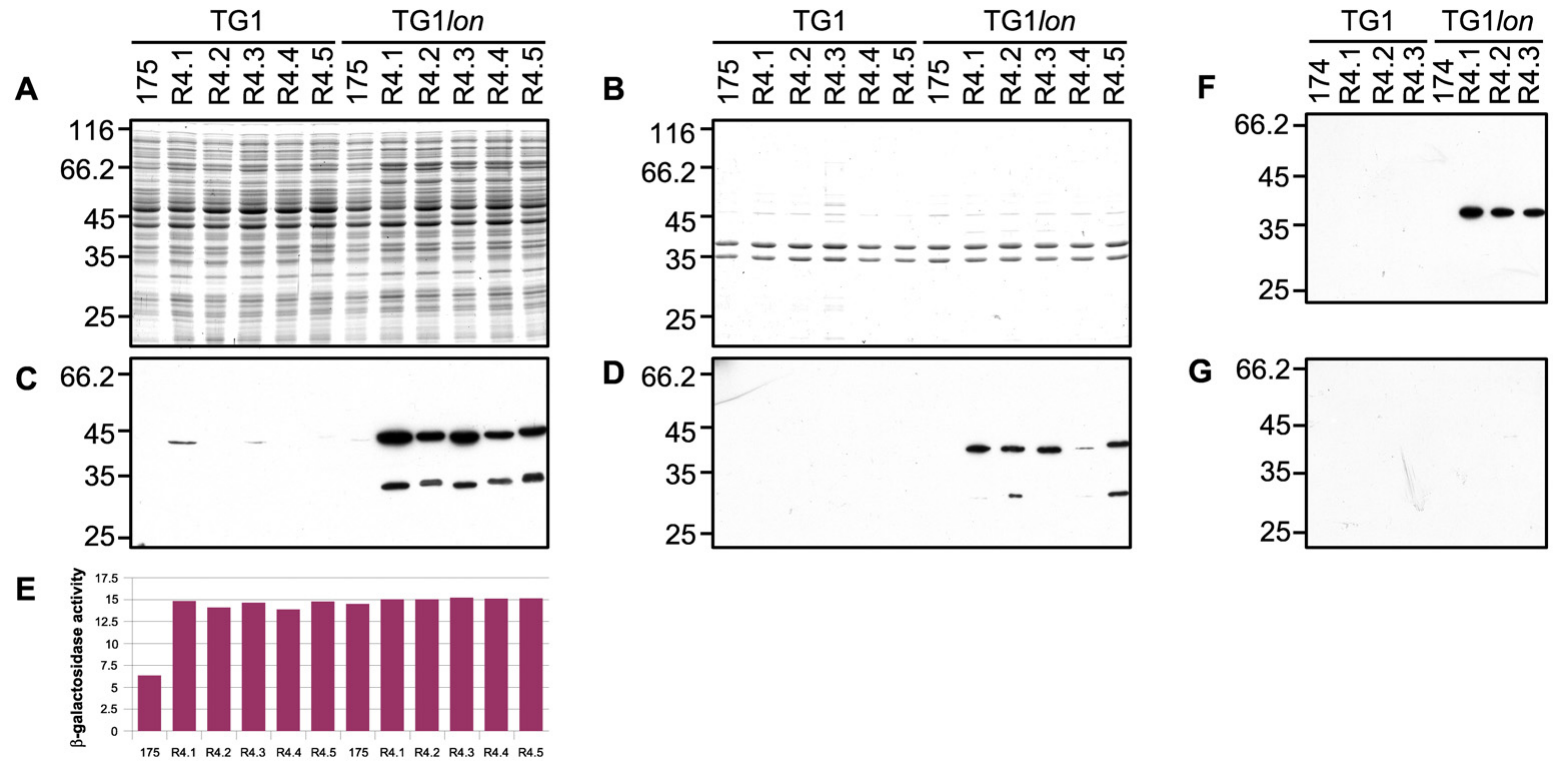
**Expression of scFv-α fusions in TG1 and TG1lon strains **SDS-PAGE of soluble (5 μl : A, C & F) and insoluble extracts (5 μl ; B, D & G) of the five best clones of scFv 225.28S (A, B, C & D) and scFv HuLys11 (F & G) expressed in TG1 and TG1*lon*. Proteins were revealed either by Coomassie blue staining (A & B) or by western blot (C, D, F & G) using an HRP-coupled anti-polyHistidine monoclonal antibody (Sigma A7058) and detected with a commercial detection kit (Pierce Supersignal West Pico kit #14079). In panel E, the same soluble extracts than in C were tested for βgal activity.

To determine whether the expression levels of the mutant scFv were higher than those of the wild type protein, soluble and insoluble extracts were prepared and analyzed (Fig. 7). No protein was detectable by western blot in the case of the wild type scFv225.28S or mutants R4.2 and R4.4, and only a faint band was visible in the soluble extracts of the other clones (R4.1, R4.3 and R4.5. Fig. 7C &7D). This showed that indeed, as expected from the βgal levels measured in Figure 5, some of the mutants were expressed at a higher level in the cytoplasm than the wild type but that most of the scFv was degraded by the host proteases.

To test this hypothesis, we expressed the clones in a TG1 strain deficient in the cytoplasmic Lon protease. This protease has been shown to be involved in the degradation of many recombinant proteins in *E. coli *[33]. When expressed in such a strain, the wild type scFv225.28S was detected in the soluble fraction, albeit at a very low level (Fig. 7C), and no scFv was present in the insoluble fraction. All the evolved scFv were expressed at a much higher level than the wild type, mainly as soluble proteins (Fig. 7C &7D), showing that the scFv225.28S proteins were predominantly degraded by the Lon protease *in vivo*. The same type of result was obtained with scFv HuLys11 since the wild type scFv was not detected in any of the extracts, and the evolved scFv were detected in the soluble fraction of the Lon deficient strain (Fig. 7F&7G). The Lon protease is not the only protease present in the *E. coli *cytoplasm [34] and in its absence some degradation of the fusion is still present as shown by the additional band at about 30 kDa in Fig. 7C &7D. This molecular weight is consistent with a degradation site located between the scFv and the α-peptide and could be one of the sources of the free α-peptide liberated in the cell cytoplasm (see below). It should be noted that in the reported cases of the successful isolation of soluble variants of aggregating proteins using the GFP system [25,26], the authors used an *E. coli *B strain, naturally deficient in the Lon protease [33,35].

The soluble extracts of scFv225.28S and its mutants were tested for the presence of βgal activity (Fig. 7E). The results obtained in strain TG1 were comparable, as expected, to those obtained previously (Fig. 5). There is however no clear correlation between the soluble expression level and the βgal activity since clone R4.1, which is expressed at the highest level, did not give a higher signal than the non-detected clones, R4.2 and R4.4. In the Lon-deficient strain, all the clones, including the wild type scFv225.28S, gave the same signal despite the fact that the wild type scFv was only barely detectable in the extract. These results showed that there was no correlation between the soluble expression level of the fusion protein and the βgal signal. The most likely explanation of this phenomenon is that the βgal signal is not due to the fusion protein detected by western blot but to some α-peptide released from the fusion by the host proteases. This free α-peptide is presumably also much more efficient for βgal complementation than the fusion protein since it may penetrate the tunnel present in M15 protein [36] at a much faster rate.

### Unfused scFv characterization

**Figure 8:**
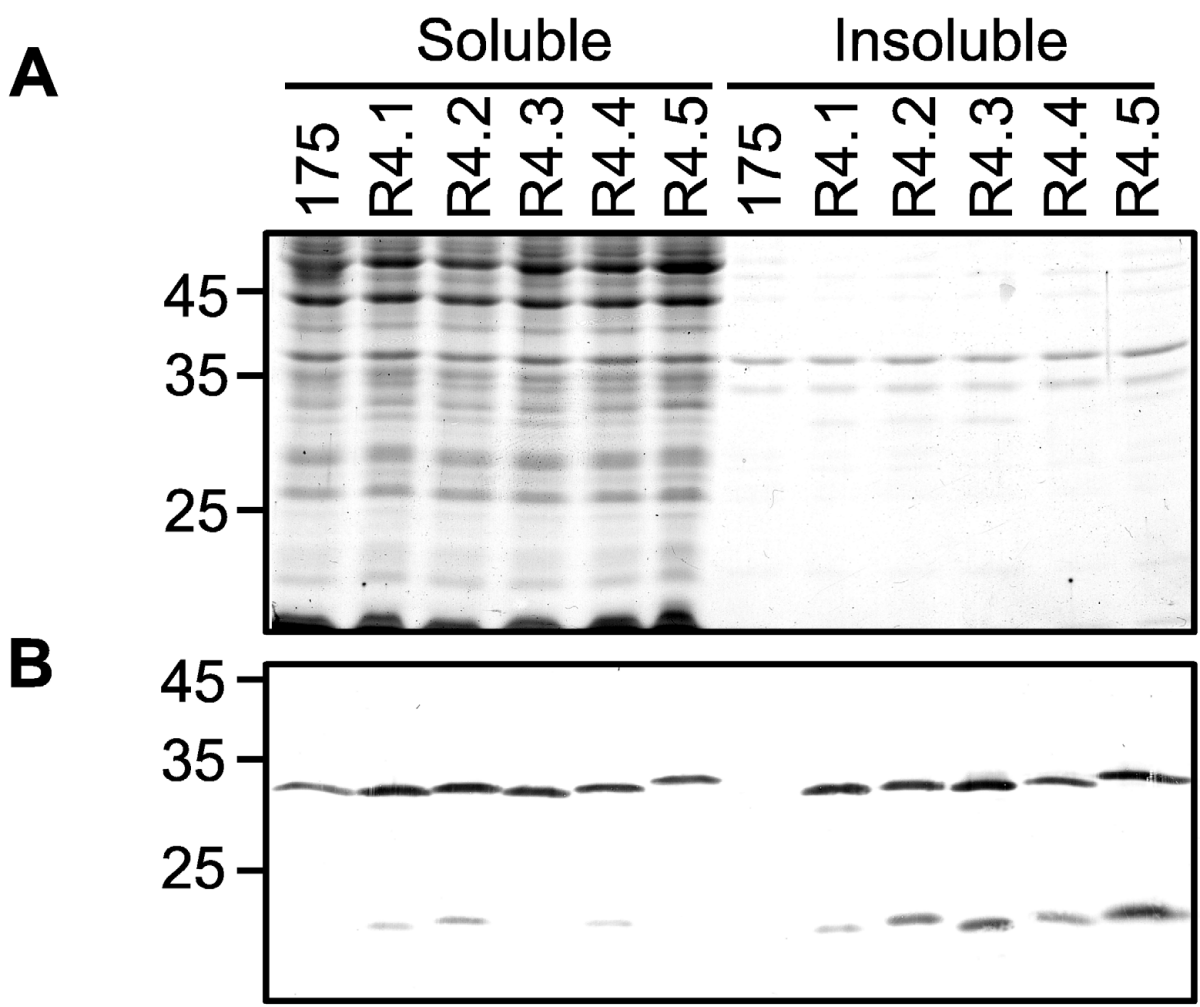
**Cytoplasmic expression of unfused scFv **After cloning in plasmid pPM210, scFv225.28S and mutants were expressed in strain TG1*lon*. 5 μl of soluble and insoluble extracts were analyzed by Coomassie blue staining (A) and western blot (B) using the 9E10 anti-*c*-myc monoclonal antibody followed by an anti-mouse alkaline phosphatase-conjugated antibody and detected with the chromogenic substrate BCIP/NBT.

The previous results were obtained with the scFv fused to the α-peptide. Since it has been shown in several systems that fusion to a partner may influence the fate of a protein [37-40], we next examined the expression level of scFv225.28S and its mutants as unfused proteins. After cloning in plasmid pPM210, the scFv were expressed in the Lon-deficient TG1 strain. The wild type scFv225.28S was only detected in the soluble fraction (Fig. 8) and the mutants were present in both the soluble and insoluble fractions. All the mutant were expressed at a higher level than the wild type scFv, but the increase in expression was less pronounced than when the proteins were fused to the α-peptide (Fig. 7). This was however mainly due to the fact that the wild type scFv225.28S was expressed at a higher level when not fused to the α-peptide because the fusion was degraded by the host degradation machinery as previously described for other βgal fusions [37].

We next compared the expression level of the best isolated clone (scFv225.28S R4.1) in the periplasm and in the cytoplasm. It must be noted that the two vectors used, pPM210 and pAB1, are derived from the same pUC119 plasmid and that in both cases the scFv gene is under the control of the *lac *promoter. The scFv expressed by the two plasmids are exactly the same except for the *pel*B leader peptide present at the N-terminal extremity of the scFv expressed in the periplasm. After cleavage of this signal sequence, the two scFv only differ by an additional N-terminal Met-Ala dipeptide present in the scFv produced in the cytoplasm.

**Figure 9:**
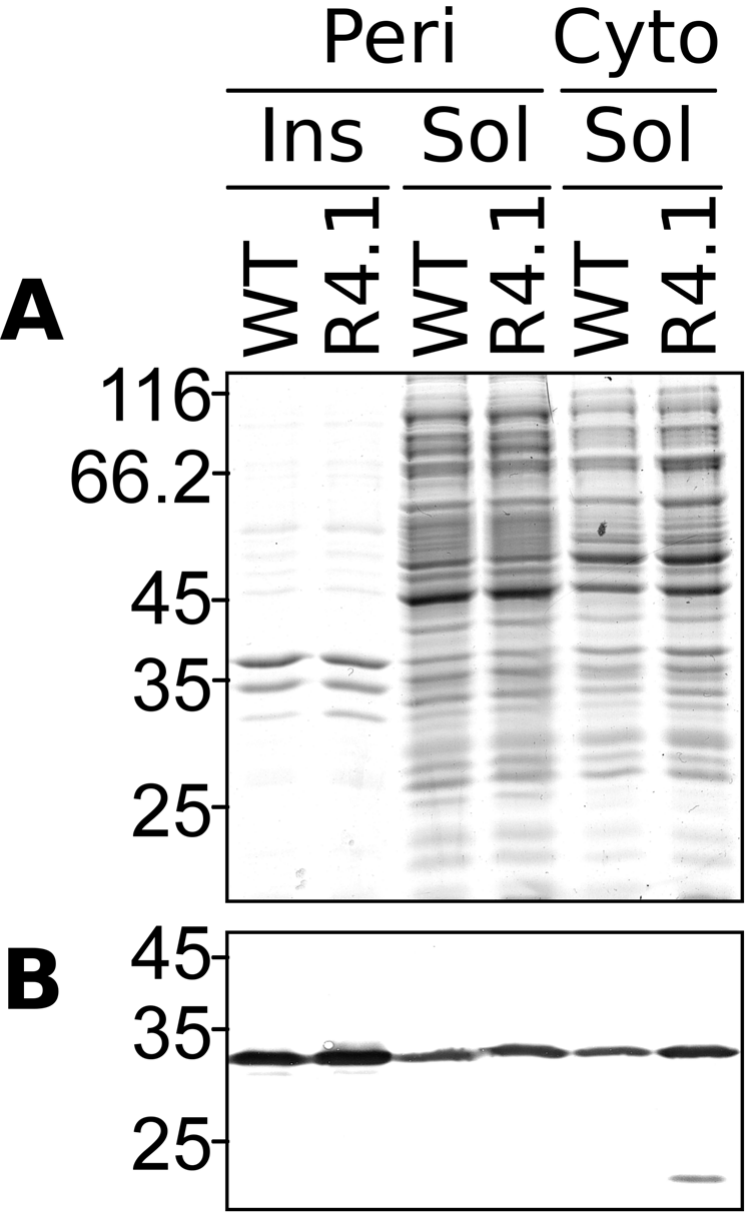
**Comparison of cytoplasmic and periplasmic soluble expression **Genes of scFv225.28S and its mutant R4.1 were cloned in plasmid pAB1 [28]. In this plasmid, scFv genes are expressed under the control of the *lac *promoter with the *pel*B signal sequence fused at their N-terminal extremity in order to target protein to the periplasm. Soluble and insoluble extracts were prepared from strain TG1 and were analyzed by Coomassie blue staining (A) and western blot (B) as in Figure 8. The two last extracts (Cyto) are those analyzed in lanes 1 and 2 of Figure 8 (soluble cytoplasmic extracts of scFv225.28S and R4.1, cloned in pPM210 and expressed in TG1*lon*).

As shown in Figure 9, the soluble expression level of mutant R4.1 is higher both in the periplasm and in the cytoplasm than that of the wild type scFv225.28S. In addition, the soluble cytoplasmic expression level of the evolved R4.1 clone is higher than the wild type periplasmic expression level. This suggests that evolving scFv for cytoplasmic expression is a valuable approach for increasing the production of scFv in *E. coli *even if in this case it is only a twofold increase much lower than in previously reported cases [28].

**Figure 10:**
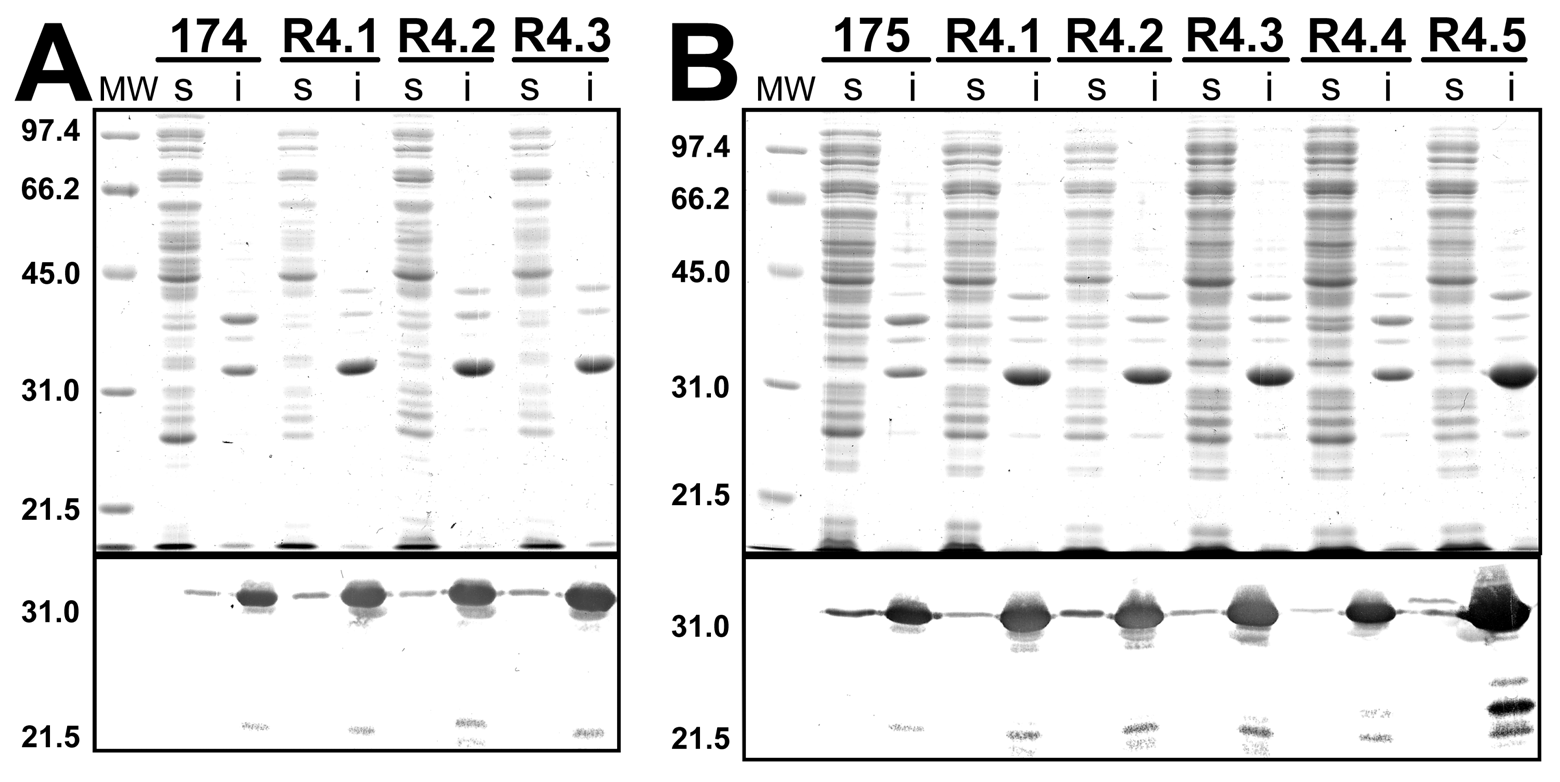
**Cytoplasmic expression under the T7 promoter **SDS-PAGE of soluble and insoluble extracts (5 μl) of the best clones isolated after four rounds of mutagenesis, cloned in the pET23NN plasmid. Proteins were revealed either by Coomassie staining (top) or by western blot using the 9E10 anti-c-myc monoclonal antibody followed by an anti-mouse alkaline phosphatase-conjugated antibody and detected with the chromogenic substrate BCIP/NBT. A) HuLys11 scFv and mutants; B) 225.28S scFv and mutants.

In an attempt to increase further the cytoplasmic expression, the genes were cloned under the control of the strong T7 promoter and expressed in strain BL21(DE3)pLysS [41], which is naturally deficient in the Lon protease [33,35]. As seen in Figure 10, the mutant proteins were expressed at much higher levels than the wild type. This is particularly the case for scFv225.28S (Fig. 10B). To further characterize the mutant proteins, soluble and insoluble protein fractions were prepared. As shown in Figure 10, the increase in expression was due to an increase in the insoluble fraction. In all cases, the amount of soluble protein produced by the mutants was comparable or even lower than that produced by the wild type. This was true both for HuLys11 and 225.28S.

## Discussion

In this report, we used the α-complementation assay as a probe to detect soluble scFv expression in *E. coli *cytoplasm [21]. The system was used to evolve two scFv to increase their expression levels. After four rounds of mutation and selection, we were able to select for scFv fusions giving high βgal activity *in vivo *and *in vitro*.

Characterization of these mutant proteins showed that they were expressed at higher levels in the cytoplasm than the wild type scFv. The proteins were however quickly degraded in the cell cytoplasm, and only a faint band was detected in the soluble fraction and no protein at all in the insoluble fraction (Fig. 7). This is not surprising for scFv expressed in the reducing environment of the cytoplasm since the lack of the two disulfide bonds results in only marginally stable proteins quickly degraded by the host proteases [42]. Since it has been shown that the Lon protease is involved in the degradation of many recombinant proteins in *E. coli *[33], we expressed the scFv in an isogenic strain deficient in this protease. Under those conditions, the mutant scFv were expressed at a much higher level than the wild type scFv. This was true for both scFv, HuLys11 and 225.28S.

This increase in soluble cytoplamic production of the scFv-α fusion was however not correlated with the βgal activity (Fig. 7). For example, clone scFv225.28S R4.1 and R4.2, which were expressed at a very different soluble level (Fig. 7C), gave the same βgal signal (Fig. 5 &7E). This indicates that most, if not all, of the βgal activity present in the cell was not due to the scFv-α-peptide fusion but to some free α-peptide released from the fusion by the host proteases. This means that the α-complementation assay does not sense the scFv-α expression level, as hypothesized, but rather the amount of free α-peptide in the cell cytoplasm. This could also explain why the system was not able to detect differences between the clones in a *lon *background (Fig. 7E).

**Figure 11:**
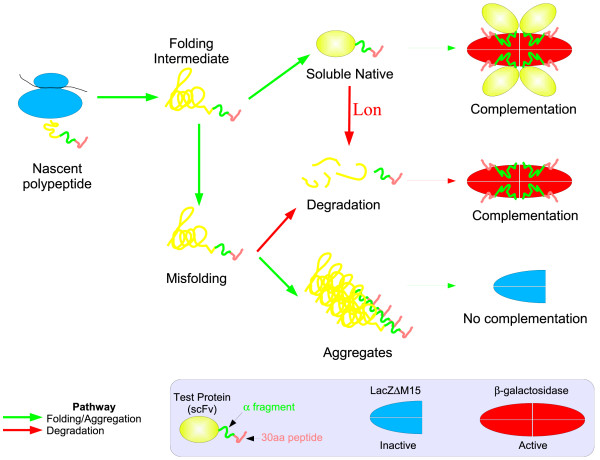
**Schematic representation of the possible in vivo fates of scFv-α protein fusion **Schematic representation of folding, aggregation and degradation processes. In the scheme presented, the newly translated protein (left) is assumed to proceed through two alternative pathways: either it folds to give the folded protein (soluble native) or it evolves via a side reaction leading to a misfolded protein (misfolding). A second kinetic competition is thought to occur between degradation and aggregation of the misfolded protein. Green arrows represent the kinetic competition between folding and aggregation [44] on which the soluble reporter assays are based [18]. Red arrows represent an additional pathway leading to degradation [43] and release of free α-peptide in the cytoplasm.

Recently, Betton and collaborators [43] presented a model of the possible *in vivo *competition between folding, aggregation and degradation. Although this model was presented in the case of periplasmic proteins it might also apply in the cytoplasm. The possible model and the results for *in vivo *βgal complementation are shown in Figure 11. The "classical" competition between aggregation and folding is represented by the green arrows at the top of Figure 11. The scFv, emerging from the ribosome, will fold into folding intermediates. A folding intermediate may fold into a soluble native conformation, or may misfold, leading to aggregation. Such a model has been extensively studied *in vivo *and *in vitro *[44-47]. In this model, an increase in α-complementation would result in an increase in soluble protein expression and is the basis of the tag-based systems to detect and evolve soluble proteins [18]. As proposed by Betton and collaborators [43], there is however a third pathway, leading to protein degradation, in kinetic competition with aggregation. When the proteins are expressed at a low level under the lac promoter, most of the protein ends up as degraded peptide fragments. However, since aggregation is a high order kinetics, this is favored over degradation under the high transcription rate due to the T7 promoter. This would not be the case if degradation had not originated from the same misfolded conformation as the aggregated protein. However, since the soluble expression levels of the scFv were higher in a *lon *strain, we must also admit that there is a supplementary pathway involving the Lon protease and leading from soluble fusion to degraded protein as suggested by Parsell and Sauer [42].

There is a striking difference between the properties of the fused and the unfused scFv. Indeed, in the structural context of the selection (as fusion to the α-peptide), all the scFv, albeit highly sensitive to degradation, were mainly expressed as soluble proteins. This is particularly the case of HuLys11 for which no insoluble protein was detected in Fig. 7G. As unfused scFv, all the clones were, however, mainly found in the insoluble fraction, particularly when expressed using the strong T7 promoter (Fig. 10) but also when we used the same promoter than during the selection process (Fig. 8). This shows that interactions could take place between the scFv and the fused α-peptide either during or after folding of the fusion protein. This may explain why all the mutations where localized on the surface of HuLys11 (Fig. 6) since these residues are more likely to take part in such an interaction. This is particularly the case of the H46 mutation, which is present in all three isolated mutants. In this case a hydrophilic and charged residue (Glu) is replaced by an hydrophobic Gly. As the α-peptide and the 30 aminoacid peptide fused to the scFv contain some highly hydrophobic patches of residues (data not shown), this increase in the hydrophobicity of the scFv surface may favor interaction between the two partners that could enhance the solubility of the fusion by a chaperone-like effect, as proposed for other fusions [48]. When the scFv is expressed alone, without the C-terminal α peptide, increasing the hydrophobicity of the surface residues could result in an increase in aggregation [49], as noted in our case (Fig. 8 &10).

Finally, another problem during the selection originated in the presence of the 30 aminoacid peptide at the end of the α-peptide. As we showed in the case of the model antibody scFv13, this peptide is needed to obtain good correlation between protein soluble expression levels and *in vivo *α-complementation since the introduction of a stop codon at the end of the α-peptide resulted in a very strong complementation with all the scFv, even the wild type (clone 173S1 in Fig. 2 &3). This may explain why the selection was biased towards degraded C-terminal α-peptide. Indeed, Figure 3 shows that the role of the additional 30 aminoacid long C-terminal peptide is to decrease the expression level of the fusion in order not to saturate the detection system with the wild type scFv. This decrease in the expression level is presumably accomplished through targeting of the fusion to the cell degradation systems, resulting in a rapid conversion of the fusion to free α-peptide and in a bias in the selection procedure towards degradation and thus aggregation. It must be noted that Schwalbach and collaborators [50] recently selected a mutant scFv using the GFP system, and that despite an increase in GFP activity, they did not notice any increase in soluble protein expression but rather an increase in protein aggregation. They also noticed that protease degradation of the fusion released free GFP in the cytoplasm, particularly when cells were induced for long periods, that is, the conditions used during the selection process. It should be noted that the authors used a *lon*+ strain for the selection instead of the *E. coli *B *lon*- strain used by other authors [25,26,35]. These failures may be due, in the specific case of cytoplasmic scFv expression, to the lack of disulfide bond formation, leading to marginally stable reduced scFv, quickly degraded by the cell proteases [42].

Despite the difficulties associated with cytoplasmic scFv expression, some scFv have been previously evolved for folding under reducing conditions [28,51]. The systems used relied on the binding activity of the scFv molecule, avoiding the problem of protein degradation since degraded antibody cannot bind to its antigen anymore. It would thus be more appropriate to use yeast or bacterial two-hybrid systems to evolve scFv [52,53]. The use of such systems could also avoid the selection of mutations that modify or abolish the antigen-binding properties of the scFv as is presumably the case in our selection since several of the mutations are located in the CDR loops of the antibody fragments (see additional files 4 and 2, HuLys11aa.txt and 22528Saa.txt). Another possibility could be to use *E. coli *protease-deficient strains to limit protein degradation during the selection, but the results shown in Fig. 7E demonstrate that even in such a strain there is no correlation between the soluble expression level and the βgal activity present in the cell.

## Conclusions

In this report, we used the α-complementation assay as a probe to detect soluble scFv expression in *E. coli *cytoplasm. The system was used to evolve two scFv in order to increase their expression levels. After four rounds of mutation and selection, we were able to select for scFv fusion giving a high βgal activity *in vivo *and *in vitro*.

Characterization of these mutant proteins showed that their expression levels were much higher in the cytoplasm than those of the wild type scFv, particularly in a Lon-deficient strain. There was however no correlation between the βgal activity present in the cell and the soluble expression level of the scFv, showing that the βgal signal is presumably due to some free α-peptide released from the fusion protein by the host proteases and not to the non-degraded soluble scFv-α fusion.

## Methods

### Media, plasmids and bacterial strains

MacConkey agar, M9 and LB media were previously described [30]. Strain TG1 is *E. coli *K-12, [F' *traD*36 *lac*I^q ^Δ(*lac*Z)M15 *pro*A^+^B^+^] *sup*E44 Δ(*hsd*M-*mcr*B)5 *thi *Δ(*lac*-*pro*AB). CAG626F' is *E. coli *K-12, [F' *lac*I^q^Δ(*lac*Z)*M*15 *zzf*::mini-*Tn*10(Kan^R^)*pro*A^+^B^+^] *lac*Z(am) *pho*(am) *lon trp*(am) *tyrT *[*supC*(ts)] *rpsL mal*(am) [54].

TG1*lon *strain was constructed by P1 transduction. First, *zaj*-*3054::Tn*10, located at 9.95 minutes, was inserted near to the *lon *gene in strain CAG626F' by P1 transduction from CAG12017 [55]. In a second step, *lon *and *zaj*-*3054::Tn*10 were co-transduced in TG1 using P1 phage. The introduction of the *lon *mutation was verified by comparing the resistance to UV irradiation and to nitrofurantoin of TG1 and TG1*lon*.

Plasmid pPM170 for the cytoplasmic expression of scFv fused to the α-fragment of βgal was constructed as follows (Fig. 1). A fragment containing a Shine Dalgarno sequence followed by a *Nco*I site containing the ATG initiator was obtained by PCR amplification of pPM160 [28] with pTrpFOR (CGGGAATAAGCTTCAACGCCAG) and EcoAlpha.for (GTGAATGAATTCGAATGGTGATGATGG) primers. The underlined sequence corresponds to an *Eco*RI site designed in order to get in frame fusion of the scFv with the α-fragment of βgal present in pUC119 when the fragment was cloned in the *Eco*RI site of pUC119. The amplified fragment also contained an *Hpa*I site located 34 bp upstream from the ATG initiator of the scFv gene. This PCR band was digested with *Hpa*I and *EcoR*I enzymes and ligated with *Hinc*II-*Eco*RI-digested pUC119 plasmid, leading to the pPM170 plasmid.

Plasmid pAB1 for the periplasmic expression of scFv under the *lac *promoter has been described previously [28]. Plasmid pPM210 is identical to pPM170 except for the presence of a stop codon between the scFv and the α-fragment of βgal. It was obtained by cloning the *Nco*I-*Eco*RI fragment of pAB1 in the *Nco*I and *Eco*RI sites of pPM170. scFv cloned in pPM210 were transcribed from a *lac *promoter and tagged at their C-terminal extremity by both a c-myc and a polyhistidine flags.

Plasmid pET23NN was designed to easily clone *Nco*I-*Not*I fragment containing scFv from pPM170-derived plasmids under the T7 promoter with C-terminal c-myc and His6 tags. pET23d(+) obtained from Novagen was first digested with *Xho*I enzyme, filled-in with T4 DNA polymerase then digested with *Nco*I. This fragment was ligated with a *Nco*I-*Eco*RI(filled) fragment excised from the pAB1 plasmid [28]. The resulting plasmid contained a T7 promoter, followed by a *Nco*I site containing the ATG initiator, a *Not*I site followed by a c-myc and a His6 tag [same sequence as pPM170 (Fig. 1) but with a stop codon before the *Eco*RI site]. Due to the removal of a T, presumably during T4 DNA polymerase treatment, ligation between the filled *EcoR*I and *Xho*I sites reconstituted the *EcoR*I site (GAATTCGAG instead of GAATTTCGAG).

### Error-prone mutagenesis

The conditions used are those described to obtain 0.2% mutations with p(AT->NN) = p(GC->NN) and p(AT->GC) = p(AT->TA) for ten duplications [28,56]. The amplified band was digested with *Nco*I and *Not*I and cloned in pPM170. The ligation was transformed in TG1 by electroporation. Bacteria were plated on MacConkey lactose and M9 lactose agar plates supplemented with 100 μg ml^-1 ^ampicillin, 1 mM isopropyl-β-D-thiogalactopyranoside (IPTG) and 1 μg ml^-1 ^of vitamin B1 in the case of the M9 plates.

### Preparation of bacterial extracts

Expression under the T7 promoter was conducted essentially as described except that LB was used instead of M9ZB [41]. Freshly transformed cells were grown in LB supplemented with 100 μg ml^-1 ^ampicillin and 20 μg ml^-1 ^chloramphenicol until an OD_600 _of about 1 and kept at 4°C overnight. In the morning, the cells were diluted to an OD_600 _of 0.1 in the same medium and incubated with shaking for 2 hours at 37°C, then 0.4 mM IPTG was added and the culture continued for 3 hours at 30°C. The cultures were cooled down on ice for 5 to 30 minutes, then centrifuged and resuspended at a concentration of 40 OD_600 _in 50 mM Tris-HCl (pH 8.0), 2 mM EDTA. Cells were lysed by freezing/thawing followed by a brief sonication. The insoluble fraction was collected by centrifugation (30 minutes at 17 500 g), washed once, then resuspended in the same volume of buffer.

For expression under the lactose promoter (plasmid pPM170), an overnight culture in LB supplemented with 100 μg ml^-1 ^ampicillin and 1% glucose was diluted to a final OD_600 _of 0.1 in LB supplemented with 100 μg ml^-1 ^ampicillin and incubated with shaking for 2 hours at 37°C, then 1 mM IPTG was added and the culture continued for 3 hours at 30°C. Cytoplasmic soluble extracts were prepared as follows. 16 OD of culture were cooled down on ice for 5 to 30 minutes, then centrifuged and resuspended in 400 μl of 20 mM Tris-HCl (pH 8.0), 0.7 M sucrose. Hen egg-white lysozyme at a 0.1 mg ml^-1 ^concentration was added and the extract incubated 2 minutes on ice. 800 μl of a cold solution of 1.5 mM EDTA were added slowly within ten minutes. Extract were kept 30 minutes on ice then sonicated 20 s. The insoluble fraction was collected by centrifugation (30 minutes at 17 500 g), washed once, then resuspended in the same volume of buffer than the soluble fraction (1.2 ml).

### Measurement of βgal activity

Cells were grown under the same conditions as those described in "Preparation of bacterial extracts" for the lactose promoter. Five hundred μl of cells were lysed using SDS/chloroform and βgal activity determined and expressed as described in Miller [30]. For Figure 5 and 7, we also prepared soluble extracts using the protocol described in "Preparation of bacterial extracts" and βgal activity was determined as above.

## Authors' contributions

PP carried out some of the mutant characterization and participated in manuscript preparation. PM conceived of the study, performed the mutagenesis and selection experiments, and participated in manuscript preparation. All authors read and approved the final manuscript.

## Supplementary Material

Additional File 1 DNA sequences of 225.28S and mutants scFv. The position of the six CDRs are indicated as H1, H2 and H3 for the VH and L1, L2 and L3 for the VL. The scFv 225.28S translated sequence is shown above the DNA. Proteins are numbered according to Kabat [57] Click here for file

Additional File 2 Aminoacid sequences of 225.28S and mutants scFv. The position of the six CDRs are indicated as H1, H2 and H3 for the VH and L1, L2 and L3 for the VL. Proteins are numbered according to Kabat [57]
Click here for file

Additional File 3 DNA sequences of HuLys11 and mutants scFv. The position of the six CDRs are indicated as H1, H2 and H3 for the VH and L1, L2 and L3 for the VL. The scFv HuLys11 translated sequence is shown above the DNA. Proteins are numbered according to Kabat[57] Click here for file

Additional File 4 Aminoacid sequences of HuLys11 and mutants scFv. The position of the six CDRs are indicated as H1, H2 and H3 for the VH and L1, L2 and L3 for the VL. Proteins are numbered according to Kabat [57]. The secondary structure of the protein, as indicated in the header of the PDB file 1BVK, is summarized above the sequence (H: helix; E: strand). Click here for file
